# A reconfigured Kennedy pathway which promotes efficient accumulation of medium‐chain fatty acids in leaf oils

**DOI:** 10.1111/pbi.12724

**Published:** 2017-05-03

**Authors:** Kyle B. Reynolds, Matthew C. Taylor, Darren P. Cullerne, Christopher L. Blanchard, Craig C. Wood, Surinder P. Singh, James R. Petrie

**Affiliations:** ^1^ Commonwealth Scientific and Industrial Research Organization, Agriculture and Food Acton ACT Australia; ^2^ Department of Primary Industries Graham Centre for Agricultural Innovation Charles Sturt University Wagga Wagga NSW Australia; ^3^ ARC Industrial Transformation Training Centre for Functional Grains Charles Sturt University Wagga Wagga NSW Australia; ^4^ Commonwealth Scientific and Industrial Research Organization Land and Water Acton ACT Australia; ^5^ School of Environmental and Life Sciences University of Newcastle Newcastle NSW Australia

**Keywords:** plant biomass, plant oils, triacylglycerol, WRINKLED, *Nicotiana*, DGAT, GPAT9, medium chain, fatty acid

## Abstract

Medium‐chain fatty acids (MCFA, C6‐14 fatty acids) are an ideal feedstock for biodiesel and broader oleochemicals. In recent decades, several studies have used transgenic engineering to produce MCFA in seeds oils, although these modifications result in unbalance membrane lipid profiles that impair oil yields and agronomic performance. Given the ability to engineer nonseed organs to produce oils, we have previously demonstrated that MCFA profiles can be produced in leaves, but this also results in unbalanced membrane lipid profiles and undesirable chlorosis and cell death. Here we demonstrate that the introduction of a diacylglycerol acyltransferase from oil palm, *EgDGAT1*, was necessary to channel nascent MCFA directly into leaf oils and therefore bypassing MCFA residing in membrane lipids. This pathway resulted in increased flux towards MCFA rich leaf oils, reduced MCFA in leaf membrane lipids and, crucially, the alleviation of chlorosis. Deep sequencing of African oil palm (*Elaeis guineensis*) and coconut palm (*Cocos nucifera*) generated candidate genes of interest, which were then tested for their ability to improve oil accumulation. Thioesterases were explored for the production of lauric acid (C12:0) and myristic (C14:0). The thioesterases from *Umbellularia californica* and *Cinnamomum camphora* produced a total of 52% C12:0 and 40% C14:0, respectively, in transient leaf assays. This study demonstrated that the introduction of a complete acyl‐CoA‐dependent pathway for the synthesis of MFCA‐rich oils avoided disturbing membrane homoeostasis and cell death phenotypes. This study outlines a transgenic strategy for the engineering of biomass crops with high levels of MCFA rich leaf oils.

## Introduction

Over recent years, the global production of vegetable oils has experienced constant growth, with over 179 million metric tons (MMT) being produced in 2015 (OECD/FAO, 2015), with the four major oil production crops being oil palm, soya bean, canola and sunflower. An important component of global oil consumption is medium‐chain fatty acids (MCFA), here defined as fatty acids in the range of 6–14 carbons in length. In addition, their application within the food industry MCFAs is an ideal source for biodiesel and also for a wide range of oleochemical feedstocks including pharmaceuticals, personal care products, lubricants and detergents (Arkcoll, 1988; Basiron and Weng, 2004). Currently, the predominant crop sources of MCFA‐enriched oils are coconut palm and oil palm (both palm oil and palm kernel oil) (Arkcoll, 1988). The production of these crops is limited to tropical and subtropical climates (Arkcoll, 1988; Basiron, 2007; Basiron and Weng, 2004; Kumar, 2011; Laureles *et al*., 2002). It has been projected that the global demand for vegetable oils will increase to approximately 240 MMT in 2050 (Corley, 2009), approximately 50% more than what is currently being produced. The development of new crops that can produce MCFA‐enriched oils in temperate climates has been proposed (Dehesh, 2001; Eccleston *et al*., 1996; Reynolds *et al*., 2015; Tjellstrom *et al*., 2013; Voelker *et al*., 1992; Wiberg *et al*., 2000) as a way to meet the growing global demand for MCFA in oleochemical production, pharmaceutical applications and personal care products.

**Figure 1:**
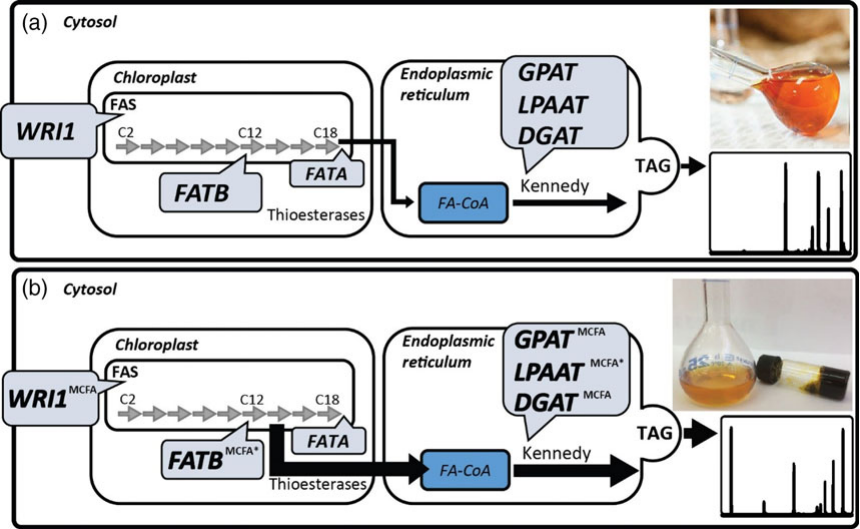
Graphical summary of the simplified TAG assembly pathway depicting the genes that have previously been used in combination for the increased production of oils (a). The oil sample represents the oil extracted from *Nicotiana tabacum* leaves with increased oil content (Vanhercke *et al*., 2014a,b), comprised of common fatty acids. The same gene families have been targets for the enrichment of the medium‐chain fatty acid (MCFA) content of oil in both previous studies (*) and in this study, denoted by ^MCFA^ (b). The oil sample (right) represents the oil extracted from vacuum infiltrated *Nicotiana benthamiana* leaves with an increased MCFA content as highlighted by the gas chromatography trace. The extracted MCFA oil remained solid until 21°C. WRI1 = WRINKLED1; FAS = fatty acid synthase; FAT = fatty acid thioesterase; GPAT = glycerol‐3‐phosphate acyltransferase; LPAAT = lysophosphatidic acid acyltransferase; DGAT = diacylglycerol acyltransferase; TAG = triacylglycerol.

Many studies have investigated the modification of seed oils to contain increased MCFA content, predominantly focused on the engineering of lauric acid (C12:0) (Eccleston and Ohlrogge, 1998; Knutzon *et al*., 1999; Voelker *et al*., 1992). In oilseeds, the engineered pathway begins with the overexpression of a specialized thioesterase (FATB) that prematurely truncates the standard fatty acid elongation cycle within the plastid allowing export into the cytoplasm. The MCFA in the cytoplasm is available for incorporation into triacylglycerols (TAG) via the endogenous oilseed pathways, with the acyl‐CoA‐dependent reactions of the Kennedy pathway (glycerol‐3‐phosphate acyltransferase (GPAT), lysophosphatidic acid acyltransferase (LPAAT) and diacylglycerol acyltransferase (DGAT)) being the preferred route to avoid incorporation onto the membrane bound lipid head groups, such as phosphatidylcholine (PC). Previous studies have investigated the incorporation of MCFA into seed oils following the coordinated over‐expression of FATB and LPAAT (Figure 1a), achieving up to 67% of seed oil (Knutzon *et al*., 1999). More recently, transcriptomic analyses have enabled the identification of new FATB and LPAAT genes from many *Cuphea* species, which have been used to both modify the fatty acid profiles and improve the incorporation of MCFA into the TAG, respectively, of transgenic *Camelina sativa* seeds (Kim *et al*., 2015a,b).

Evidence, although, has found that endogenous TAG synthesis pathways in developing oilseeds are not ideal for incorporating MCFA into TAG (Wiberg *et al*., 1997, 2000) and that newly synthesized MCFA become incorporated into membrane bound lipids, which impedes lipid flux, agronomic performance and can even result in cell death through chlorosis (Bates *et al*., 2014; Voelker *et al*., 1996). Therefore, it would seem that although MCFA can be produced in plant cells, there is a poor pathway for incorporation into seed TAG. It has also been recognized that the accumulation of unusual fatty acids in PC appears to be a bottleneck for their enriched incorporation into TAG (Bates and Browse, 2011; Reynolds *et al*., 2015). In the example of engineering ricinoleic acid into oilseeds, it has been demonstrated that the endogenous pathways need to be removed in conjunction with the ectopic expression of the specialized pathway counterpart (Adhikari *et al*., 2016; Bates and Browse, 2011; Burgal *et al*., 2008; Chen *et al*., 2016; van Erp *et al*., 2011, 2015).

Recent work has demonstrated that engineering high oil levels in plant biomass is a realistic proposition (Vanhercke *et al*., 2013, 2014a,b) with the accumulation of significant levels of TAG in *Nicotiana tabacum* leaves (up to 15%) being attained by the coordinated transgenic expression of genes normally involved in oil production in seeds (Vanhercke *et al*., 2014b). Such approaches have uncovered a synergism involving an increase the production of fatty acids in the plastid (WRINKLED1 (WRI1)), improving the assembly of fatty acids into leaf oils (DGAT) and slowing the catabolism of these oils (OLEOSIN, OLE1 (Fan *et al*., 2013; Winichayakul *et al*., 2013)); and sugar‐dependent‐1, SDP1 (Fan *et al*., 2014; Kelly *et al*., 2013; Kim *et al*., 2014; Vanhercke *et al*., 2014a). Although the production of TAG in biomass offers a new source of common vegetable oils, these new expression platforms could also be adapted to produce high levels of novel fatty acids, such as MCFA (Reynolds *et al*., 2015; Wood, 2014). Our first steps in this direction involved the overexpression of thioesterases from *Umbellularia californica*,* Cinnamomum camphora* and *Cocos nucifera* which resulted in the production of MCFA in leaf tissues (Reynolds *et al*., 2015). However, these metabolic pathways also resulted in high levels of MCFA in PC resulting in severe chlorosis and cell death (Bates *et al*., 2014; Wiberg *et al*., 2000), similar to conclusions drawn from oilseed engineering. The incorporation of MCFA into the membrane lipids of vegetative tissues is hence particularly problematic.

In this report, we extend the MCFA metabolic pathway by combining a series of previously published gene ensembles with three different DGAT1‐like genes isolated from the kernel of *Elaeis guineensis* (African oil palm). We also identified a functional GPAT9 from *C. nucifera* that was included in the metabolic pathway for improving the incorporation of MCFA into seed oils (Figure 1b). In this study, we demonstrate an improvement in MCFA utilization, which results in more efficient sequestering of MCFA in TAG, while also effectively limiting the accumulation of MCFA in membrane lipids. This nonseed approach also allows metabolic engineers to avoid some of the bottlenecks associated with oilseed engineering where the more common fatty acids are rapidly and specifically incorporated into seed oils.

## Results

### Identification of functional GPAT9 from Cocos nucifera (coconut)

*AtGPAT9* has recently been identified as being responsible for the acylation of acyl‐CoAs to the *sn*‐1 position of the glycerol‐3‐phosphate (G3P) backbone, which also demonstrated the ability to increase TAG production (Shockey *et al*., 2016; Singer *et al*., 2016). Based on the fatty acid composition of oil extracted from *C. nucifera* (coconut), it was hypothesized that a *GPAT9* from coconut may assist in boosting the MCFA content of transgenic oils. A homologous gene was identified by BLAST searching an assembled coconut endosperm transcriptome. Following isolation and sequencing of the full‐length transcript of interest, the open reading frame for the predicted *CnGPAT9* was identified. The open reading frame was used for subsequent sequence alignment with the *AtGPAT9* homolog, revealing that the sequences are ≈78% identical (Figure S1). Following multiple sequence alignment with other annotated *GPAT* sequences (Figure S2), it was confirmed that the isolated *CnGPAT9* transcript clustered with other identified and predicted *GPAT9* sequences.

**Figure 2:**
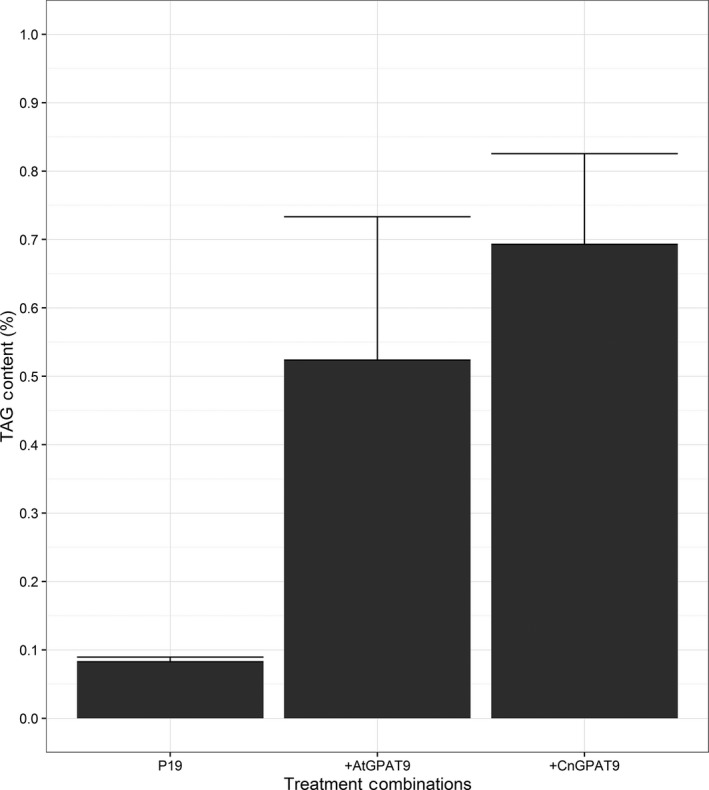
Testing the effect of glycerol‐3‐phosphate acyltransferase 9 (*GPAT9*) expression on triacylglycerol (TAG) content, comparing genes from *Arabidopsis thaliana* (*AtGPAT9*) and *Cocos nucifera* (*CnGPAT9*), determined by application of the transient *Nicotiana benthamiana* leaf expression assay (*n* = 4).

Following identification of the full‐length sequence, the candidate *CnGPAT9* was cloned into pJP3343 for testing functionality. Initially, the *CnGPAT9* was tested for functionality using the transient *N. benthamiana* infiltration assay, by testing its ability to increase TAG content, through comparison against the *AtGPAT9* as a positive control. Total lipids were extracted and analysed to determine the effect of *GPAT9* expression on TAG content (Figure 2). From comparison with the p19 alone samples (0.1 ± 0.0%), it was determined that the expression of either *AtGPAT9* (*P* = 0.05) and *CnGPAT9* (*P* = 0.01) was sufficient in leading to significant increases in the TAG content in the leaf being 0.5 ± 0.2% and 0.7 ± 0.1%, respectively. However, there was no significant differences in the increased TAG effect between either *GPAT9* candidates (*P *= 0.18). Collectively, these data illustrated that the isolated candidate sequence from coconut was a functional *GPAT9*.

### DGAT1 from oil palm promotes production of MCFA‐enriched oils

It has been previously demonstrated that MCFA‐containing oils could be produced in the leaves of *N. benthamiana* (Reynolds *et al*., 2015). Chlorosis was observed in some gene combinations following the high proportion of MCFA accumulating in the membrane lipids, especially PC. It was therefore hypothesized that the introduction of a DGAT capable of utilizing MCFA for esterification into TAG may increase the MCFA content while alleviating the chlorosis phenotype, while also increasing TAG production.

The publication of the *Elaeis guineensis* (African oil palm) transcriptome (Dussert *et al*., 2013) revealed gene candidates that may be involved in lipid synthesis pathways. These data included sequences for three predicted *DGAT1*s, which were codon optimized for expression in *N. tabacum* and synthesized by GeneArt. Although these candidates had not yet been characterized in terms of their substrate specificity, the fatty acid profile of the oils from oil palm (palm oil and palm kernel oil) (Edem, 2002) suggested that these *DGAT1* candidates may exhibit specificity for MCFA substrates. These *EgDGAT1* candidates were infiltrated in combination with *AtWRI1* (*Arabidopsis thaliana* WRINKLED1) and *CnLPAAT*, both with and without the co‐expression of *CcTE* (thioesterase from *Cinnamomum camphora*), to determine their ability to both mediate TAG production and the incorporation of MCFA into TAG. Following 5 days of expression, observations of the leaves revealed that the negative chlorosis phenotype was associated with several gene combinations (Figure S3), but correlated with the expression of the *CcTE* in particular. Further analysis revealed that the chlorosis phenotype was alleviated by the addition of *EgDGAT1.1* (a gene which is expressed in the palm kernel) when compared with *AtDGAT1*. It was hypothesized that the phenotypic improvement was due to the improved capacity of the *EgDGAT1.1* to sequester elevated levels of MCFA into TAG. Analysis of the predicted *Eg*DGAT1 genes via multiple alignments of the translated proteins (Figure S4) revealed that both the *EgDGAT1.2* and *EgDGAT1.3* isoforms may be nonfunctional due to the absence of highly conserved C‐ and N‐terminal motifs (Cao, 2011), which are responsible for the catalytic and regulatory activities of the DGAT1 enzyme, respectively (Liu *et al*., 2012; Xu *et al*., 2008).

**Figure 3:**
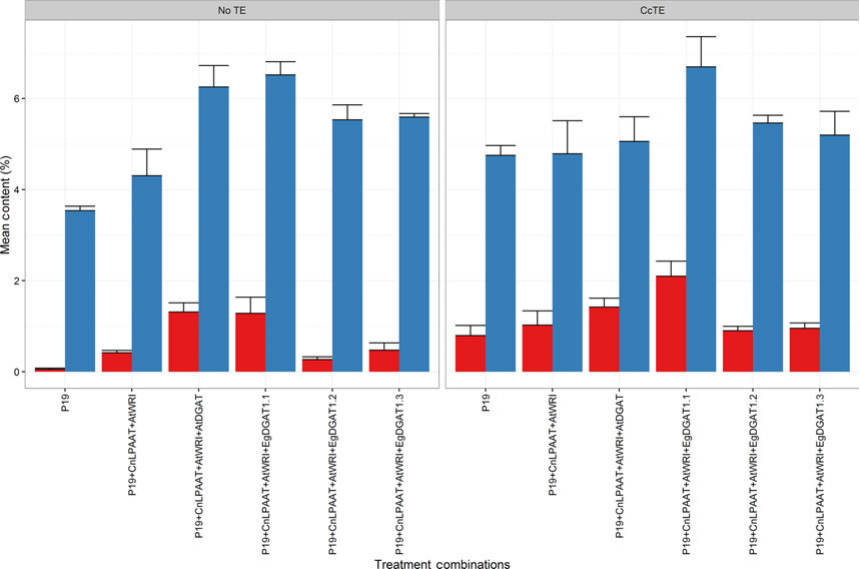
Total fatty acid (TFA; blue) and triacylglycerol (TAG; red) contents of *Nicotiana benthamiana* leaf infiltration samples (*n* = 3) were quantified. ‘CcTE’ represents the expression of the thioesterase from *Cinnamomum camphora*, whereas ‘No TE’ represents samples where *CcTE* expression was absent. *CnLPAAT* = *Cocos nucifera* lysophosphatidic acid acyltransferase; AtWRI = *Arabidopsis thaliana* WRINKLED1; AtDGAT = *A. thaliana* diacylglycerol acyltransferase 1; EgDGAT = *Elaeis guineensis* diacylglycerol acyltransferase.

Total lipids were extracted and analysed to better understand the relationship between chlorosis and particular gene combinations. The total fatty acid profile (Figure 3; blue) revealed that in the absence of *CcTE* expression, the TFA content was similar following the addition of either *AtDGAT1* or *EgDGAT1.1* (*P* = 0.23). Following the introduction of the *CcTE,* the TFA content was significantly higher for treatments including *EgDGAT1.1* than compared to *AtDGAT1* (*P* = 0.015). The same correlation was observed for TAG content (Figure 3; red). Although the TAG content was similar for *AtWRI1 *+ *AtDGAT1* and *AtWRI1 *+ *EgDGAT1.1* samples (*P* = 0.45), following the addition of *CcTE* the TAG content was significantly increased for samples expressing *EgDGAT1.1*, compared to samples expressing the *AtDGAT1* (*P* = 0.027). This result suggested that following *CcTE* expression, in the presence of *AtDGAT1*, fatty acid synthesis (FAS) was inhibited due to inefficient assembly of the MCFA into glycerolipids. Conversely, there appeared to be no inhibition of FAS following the addition of *EgDGAT1.1* (here within referred to as *EgDGAT1*) highlighted by increases in both the TFA and TAG content, implying improved incorporation efficiency for MCFAs.

**Figure 4:**
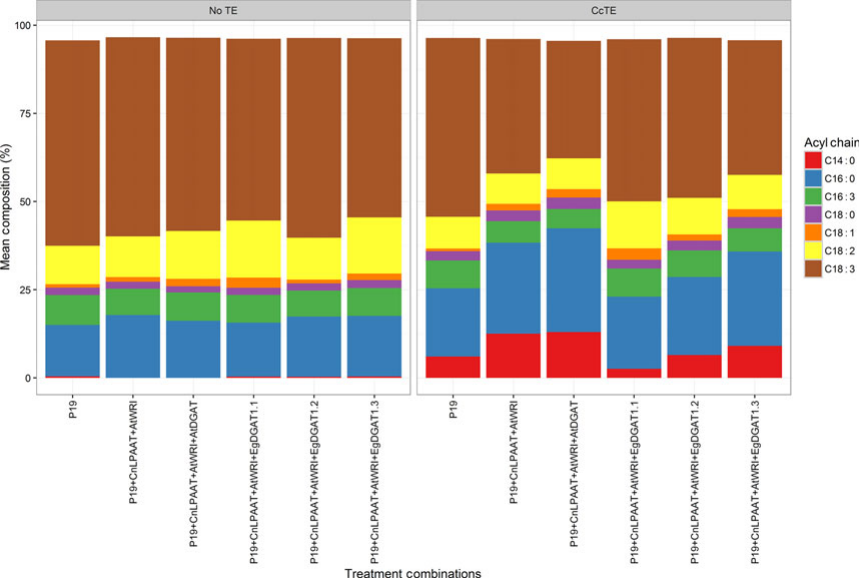
Investigating the effects of *DGAT1* co‐expression on fatty acid profile changes in the membrane lipids (phospholipids) (*n* = 3). Results in the figure represent each fatty acid as a percentage of total fatty acid methyl esters (FAME) of the phospholipid fraction only. ‘CcTE’ represents the expression of the thioesterase from *Cinnamomum camphora*. CnLPAAT = *Cocos nucifera* lysophosphatidic acid acyltransferase; AtWRI = *Arabidopsis thaliana WRINKLED1*; AtDGAT = *A. thaliana* diacylglycerol acyltransferase 1; EgDGAT = *Elaeis guineensis* diacylglycerol acyltransferase.

The fatty acid composition of the phospholipid fraction was also analysed (Figure 4). Total phospholipids were fractionated by TLC and prepared for analysis by the preparation of FAME. Analysis of the fatty acid composition of the phospholipids revealed a significant reduction in the accumulation of MCFA, particularly C14:0 and C16:0, following the over‐expression of the *EgDGAT1* (*P* = 0.008), than compared to the expression of *AtDGAT1*. This suggested that the reduced accumulation of MCFA into membrane lipids assisted in the improvement of the chlorosis phenotype.

### The reconfiguration of a Kennedy pathway for efficient MCFA accumulation

Following confirmation of the *CnGPAT9*, the *GPAT9* was used for subsequent studies that involved investigating its capability to use MCFA acyl‐CoAs as substrates for TAG assembly. The *CnGPAT9* was tested through the sequential reconfiguration of the Kennedy pathway, rebuilding the pathway for improving MCFA composition, with all infiltrations performed using *AtWRI1* to elevate fatty acid synthesis. After 5 days of gene expression, photographs of the leaves (Figure S5) were taken before the infiltrated zones were harvested and processed for the extraction of total lipids as previously described (Wood *et al*., 2009).

**Figure 5:**
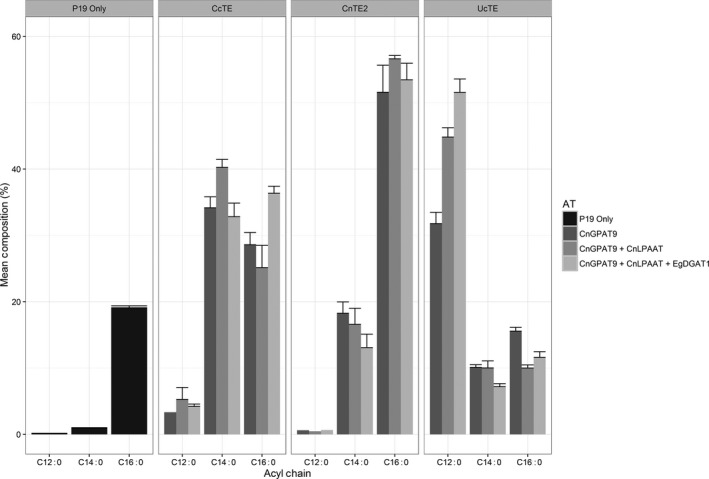
Fatty acid composition analysis of triacylglycerol (TAG), determined by the analysis of fatty acid methyl esters (FAME) via gas chromatography–flame ionization detection (GC‐FID) (*n* = 3). Each thioesterase was infiltrated in combination with *CnGPAT9* alone, *CnGPAT9* + *CnLPAAT* or *CnGPAT9* + *CnLPAAT* + *EgDGAT1*, with all treatments including expression of *AtWRI1* (*Arabidopsis thaliana WRINKLED1*). CcTE = *Cinnamomum camphora* thioesterase; CnTE2 = *Cocos nucifera* thioesterase; UcTE = *Umbellularia californica* thioesterase; *CnGPAT9* = *C. nucifera* glycerol‐3‐phosphate acyltransferase 9; *CnLPAAT* = *C. nucifera* lysophosphatidic acid acyltransferase; *EgDGAT1* = *Elaeis guineensis* diacylglycerol acyltransferase.

**Figure 6:**
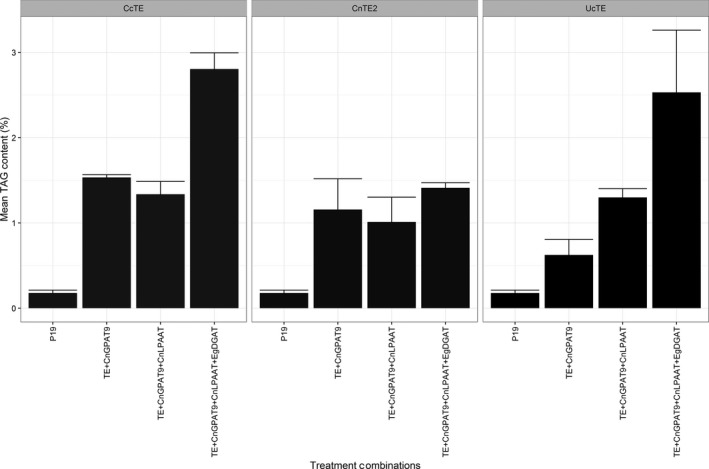
Quantified TAG content, determined by the analysis of fatty acid methyl esters (FAME) via gas chromatography–flame ionization detection (GC‐FID) (*n* = 3). The results were grouped by thioesterase expression including from *Cinnamomum camphora* (*Cc*TE), *Cocos nucifera* (*Cn*TE2) or *Umbellularia californica* (*Uc*TE). All treatments were also co‐infiltrated with WRI1 from *Arabidopsis thaliana* (*At*WRI1). *Cn*GPAT9 = *C. nucifera* glycerol‐3‐phosphate acyltransferase 9; *Cn*LPAAT = *C. nucifera* lysophosphatidic acid acyltransferase; *Eg*DGAT1 = *Elaeis guineensis* diacylglycerol acyltransferase 1.

Both the fatty acid composition of TAG and the TAG content were determined by GC‐FID (Figures 5 and 6, respectively). With the co‐expression of *UcTE*, the sequential addition of each acyltransferase resulted in both significantly increased total TAG content (*P* ≤ 0.05), and a significantly increased accumulation of C12:0 in the TAG profile (*P* ≤ 0.02). The highest level of C12:0 accumulation achieved was 51.6 ± 2.0%, which was associated with the combined expression of *UcTE *+ *AtWRI1 *+  *CnGPAT9 *+ *CnLPAAT *+ *EgDGAT1*, with a total TAG content of 2.4 ± 0.7%. It was also observed that this combination was associated with the recovery of the chlorosis phenotype, predicted to be a result of efficient sequestering of laurate into TAG, and hence the exclusion from membrane lipids. Similar results were observed with the associated co‐expression of the *CcTE*. The highest level of accumulation of C14:0 was 40.3 ± 1.2%, with the combination of *CcTE *+ *AtWRI1 *+ *CnGPAT9 *+ *CnLPAAT*, although there was no significant difference in the TAG content compared to the addition of the *CnGPAT9* only (*P* = 0.08). The greatest TAG production was achieved following the further addition of the *EgDGAT1*, with a total TAG content of 2.8 ± 0.2%. However, the fatty acid composition of TAG was altered following the additional expression of *EgDGAT1*, with both a significant reduction in C14:0 (*P* = 0.015) and a significant increase in C16:0 (*P* = 0.013) content. This shift in profile suggests that *EgDGAT1* exhibits a stronger substrate preference for C16:0 compared to C14:0. Consistent with the *UcTE* scenario, a significant improvement in the chlorotic phenotype was observed following the addition of the *EgDGAT1*. However, in the scenario of samples associated with the expression of *CnTE2*, the sequential addition of the acyltransferases did not result in any significant differences in either the fatty acid profile of TAG, or the total TAG content. This may be due to the native acyltransferases' ability to efficiently utilize the increased flux of C16:0 acyl‐CoA associated with the activity of *CnTE2*. However, the observed chlorotic phenotype suggests that efficient assembly of TAG was not occurring. Consequently, further investigations into the composition of other lipid classes, particularly the membrane lipids, were essential in understanding the occurrence of chlorosis.

**Figure 7:**
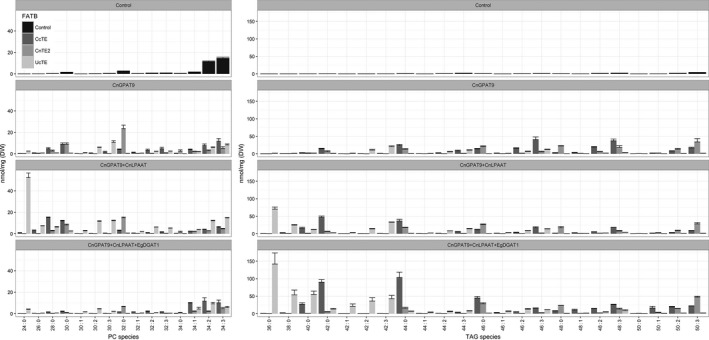
Comparative analysis of phosphatidylcholine (PC) and triacylglycerol (TAG) lipid species, associated with the expression of thioesterases from either *Cinnamomum camphora* (*CcTE*), *Cocos nucifera* (*CnTE2*) or *Umbellularia californica* (*UcTE*) (*n* = 3). ‘Control’ refers to samples that were infiltrated with p19 only. All other samples were co‐infiltrated with both p19 and *WRINKLED1* from *Arabidopsis thaliana* (*AtWRI1*). CnGPAT9 = *C. nucifera* glycerol‐3‐phosphate acyltransferase 9; CnLPAAT = *C. nucifera* lysophosphatidic acid acyltransferase; EgDGAT1 = *Elaeis guineensis* diacylglycerol acyltransferase.

Further investigations into the effects of the sequential addition of acyltransferases on the utilization of acyl‐CoAs for the assembly of MCFA‐enriched glycerolipids were performed using QQQ‐LCMS, to reveal any differences in MCFA assembly and distribution. The integrated analysis including DAG (Figure S6), PC and TAG reveals an extensive amount of information in respect to the assembly process of lipids. From the expression of *CnGPAT9*, in the background of *UcTE* + *AtWRI1* (Figure 7), it was observed that *CnGPAT9* appears to utilize C12:0 for assembly, based on the presence of PC 30:3 (C12:0 plus C18:3). It was reasoned that the *sn*‐2 position is most likely occupied by the C18:3, due to either the esterification of C12:0 to the *sn*‐1 position via *CnGPAT9* or from the absence of *CnLPAAT*. The presence of some TAG 42:3 suggests that the native *DGAT*s exhibit some capability of utilizing C12:0 for TAG assembly (12:0/18:3/12:0). Following the subsequent addition of *CnLPAAT*, a significant amount of PC 24:0 (di‐C12:0) was being produced, indicative that C12:0 was being efficiently esterified to both the *sn*‐1 and *sn*‐2 positions of the G3P backbone. However, without a *DGAT* that exhibits strong substrate preference for C12:0, most of the produced laurate remains sequestered in membrane lipids. With the further addition of the *EgDGAT1*, previously shown to be capable of utilizing MCFA for lipid assembly, a shift in laurate accumulation was observed. This shift involved the reduction MCFAs accumulating in PC, and a correlating increase production of MCFA‐enriched TAG. Most notable was the shift from PC 24:0 (without *EgDGAT1*) to the accumulation of TAG 36:0 (tri‐C12:0) (with *EgDGAT1*), highlighting that laurate was being efficiently incorporated into all three position of the G3P backbone. Significant increases were also observed for other MCFA‐enriched TAG species including TAG 38:0, TAG 40:0 and TAG 42:0. These results confirm that the expression of an appropriate *DGAT1* is essential for the efficient incorporation of the unusual fatty acids of interest (in this scenario, C12:0) into TAG. Conclusively, these results highlighted that the expression of the *EgDGAT1* effectively relieves the accumulation of laurate in PC and promotes efficient production of laurate‐enriched TAG in plant leaf lipids.

A similar pattern was also observed in the case study involving the over‐expression of *CcTE* (Figure 7). From the expression of *CnGPAT9*, in the background of *CcTE* + *AtWRI1*, it was observed that *CnGPAT9* appears to utilize C14:0 for assembly, based on the accumulation of PC 28:0 (di‐C14:0) and PC 30:0 (C14:0 plus C16:0). It appears that the native *LPAAT* genes are somewhat capable of utilizing C14:0 acyl‐CoA for lipid assembly based on the presence of PC 28:0, indicating that C14:0 was being esterified to both the *sn*‐1 and *sn*‐2 positions. Similarly, the native *DGAT*s also appear capable of utilizing C14:0 for TAG assembly, based on the production of TAG 42:0 (tri‐C14:0). However, the subsequent addition of *CnLPAAT* to the system increased utilization of C14:0 acyl‐CoA evident from the significantly increased abundance of PC 28:0, which indicated an increased efficiency of esterification to the *sn*‐2 position of the G3P backbone. This increased accumulation of MCFA was also correlated with a more severe chlorosis phenotype then compared to the *CnGPAT9* alone, most likely attributed to the increased saturation of the membrane lipids. The further addition of the *EgDGAT1* resulted in almost the complete removal of MCFA accumulation in PC. This was associated with an increased production of MCFA‐enriched TAG species, particularly TAG 40:0, TAG 42:0, TAG 44:0 and TAG 46:0, all of which include the incorporation of C14:0. It was suspected that the *EgDGAT1* exhibits a stronger preference for C16:0 than compared to C14:0, based on the higher abundance of TAG 44:0 (14:0/14:0/16:0) than TAG 42:0 (tri‐C14:0). This is supported by the similar trend seen in the changes in total fatty acid composition of TAG (Figure 5).

However, in the case study involving the over‐expression of *CnTE2* (Figure 7), the pattern was not as consistent as previously seen. From the expression of *CnGPAT9*, in the background of *CnTE2* + *AtWRI1*, it was observed that *CnGPAT9* also utilizes C16:0 for assembly, based on the accumulation of PC 32:0 (di‐C16:0). Based on the fatty acid profile of *N. benthamiana* leaves, it was expected that the native *LPAAT*s and *DGAT*s exhibit substrate specificity for the incorporation of C16:0 acyl‐CoAs into glycerolipids, evidenced from the increased production of C16:0‐enriched TAG species, through simply over‐expressing a thioesterase with C16:0 specificity. Although the subsequent additions of the *CnLPAAT* and *EgDGAT1* did not appear to significantly affect the overall TAG composition, there was significant reduction in the total MCFA accumulation in PC lipids. Importantly, the addition of the *EgDGAT1* was associated with a reduction in the degree of leaf chlorosis, although not completely recovered.

Through analysing the effect that *CnGPAT9* expression has on the fatty acid profile of both the PC and TAG lipid fractions, it was concluded that *CnGPAT9* is an important factor in contributing towards both MCFA accumulation and increasing the total production of TAG in plant leaves. In all thioesterase scenarios, the low abundance of MCFA‐containing DAG species suggests that they are actively converted to PC via the activities of either *PDCT* or *CPT* (Bates and Browse, 2011, 2012; Bates *et al*., 2012), in the absence of a MCFA‐specific *DGAT*. The addition of *EgDGAT1* changes the metabolic flux of the system, pushing MCFA towards TAG accumulation via the Kennedy pathway, and hence away from incorporation into membrane lipids via avoiding the conversion of DAG to PC.

### Modification of TAG profile through thioesterase co‐expression

With the knowledge that fatty acid thioesterases are essential factors in the fatty acid profile determination of plant seed oils (Harwood, 1996), it seems reasonable to suggest that further modifications of the fatty acid profile could be achieved through the combinatorial expression of different thioesterases. The thioesterases (*UcTE*,* CcTE* and *CnTE2*) were tested in combinations with each other using the transient *N. benthamiana* infiltration system. The thioesterases were either infiltrated alone, in pairs or in trio, with all thioesterase combinations being tested with and without the high oil background, consisting of *AtWRI1* + *CnLPAAT* + *EgDGAT1*.

**Figure 8:**
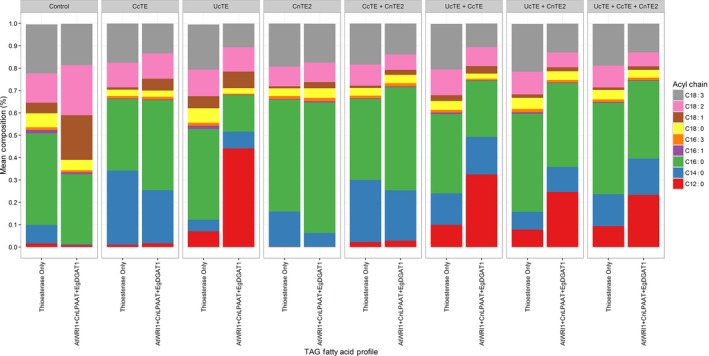
Investigating the effects of combined thioesterase expression on triacylglycerol (TAG) fatty acid composition. Results are presented as the percentage for each fatty acid, determined by GC‐FID (*n* = 4). The ‘Control’ refers to the infiltration of p19 alone. The label ‘Thioesterase Only’ refers to the expression of p19 with the respective thioesterase combination. AtWRI1 = *Arabidopsis thaliana WRINKLED1*; CnLPAAT = *C. nucifera lysophosphatidic acid acyltransferase*; EgDGAT1 = *Elaeis guineensis diacylglycerol acyltransferase*; CcTE = *Cinnamomum camphora* thioesterase; CnTE = *Cocos nucifera* thioesterase; UcTE = *Umbellularia californica* thioesterase.

To understand the contribution of each thioesterase on the assembly of TAG, the TAG composition and fatty acid profile was analysed via LC‐MS. Initially TAG was characterized by its fatty acid profile (Figure 8). With the expression of *UcTE* in the high oil background, C12:0 accounted for 48% of the TAG profile. However, significant decreases in the C12:0 content were observed, associated with significant increases in C14:0 and C16:0 following the addition of *CcTE* or *CnTE2*, respectively. Conversely, a similar pattern was observed in the scenario of *CnTE2* expression, which results in the accumulation of 58% C16:0 in TAG when expressed alone. The content of C16:0 was decreased following co‐expression of another thioesterase, correlated with increased proportions of C12:0 and C14:0 with the co‐expression of *UcTE* or *CcTE*, respectively. When the thioesterases were co‐expressed, the total MCFA content accounted for approximately 75% of the total fatty acid profile. Interestingly, this co‐ordinated expression of all three thioesterases produced a TAG profile which was similar in proportions of C12:0 (24%), C14:0 (16%) and C16:0 (34%).

The TAG profile was further analysed through determining the abundance of individual TAG species (Figure S7), which reveals more information in respect to TAG assembly and the co‐ordinated effects of the studied thioesterases on TAG composition. When *UcTE* was expressed alone, the most abundant TAG species was TAG 36:0 (tri‐laurate), with TAG 38:0, TAG 40:0, TAG 42:0, TAG 42:1, TAG 42:2 and TAG 42:3 also being highly abundant. Following the co‐expression of *CcTE* and *CnTE2,* there were significant decreases in the abundance of TAG 36:0, TAG 38:0 and unsaturated TAG 42 species. This correlated with significant increases for TAG 42:0, TAG 44:0 and TAG 46:0. The increased abundance of larger TAG species can be justified by the increased availability of C14:0 and C16:0 for incorporation into TAG, following the expression of *CcTE* and *CnTE2*. When *CcTE* was expressed alone, the most abundant TAG species was TAG 44:0, with TAG 42:0, TAG 48:3, TAG 50:2 and TAG 50:3 being almost equally abundant. With the addition of *UcTE*, increases were observed for the lower molecular weight TAG species particularly TAG 36:0, TAG 38:0 and TAG 40:0, due to the increased availability of C12:0 for TAG assembly. Alternatively, when combined with *CnTE2,* increases were observed for higher molecular weight TAG species particularly TAG 46:0, TAG 48:0 and TAG 50:3, indicative of increased availability of C16:0. When only the *CnTE2* was expressed, the most abundant TAG species were TAG 46:0, TAG 48:0, TAG 50:2 and TAG 50:3. Similar to the *CcTE* scenario, the co‐expression with *UcTE* led to an increase in the abundance of many lower molecular weight TAG species including TAG 36:0, TAG 38:0, TAG 40:0, TAG 42:0 and TAG 44:0. The addition of the *CcTE* also led to changes in the TAG composition, primarily increased abundance of TAG 40:0, TAG 42:0, TAG 44:0 and TAG 46:0. From the combinatorial expression of all three thioesterases, there was a broad range in the TAG composition, highlighting the increased availability of C12:0, C14:0 and C16:0 for the assembly of glycerolipids.

## Discussion

In the seeds of native plants, the incorporation of unusual fatty acids is almost exclusively confined to TAG and typically excluded from membrane lipids, most likely because they interfere with proper membrane functions and are often deleterious to the plant cells (Millar *et al*., 2000). A different scenario has been observed in transgenic plants that have attempted to modify the oil fatty acid profiles, such as increasing the lauric acid content (Knutzon *et al*., 1999). Although high levels of laurate accumulation in plant oils have been achieved in the seeds of transgenic canola, at ≈67%, there was a significant level of laurate being sequestered in PC during seed development (Wiberg *et al*., 1997). In this earlier transgenic canola work, *de novo* DAG containing laurate is not being efficiently converted to TAG by the resident *DGAT* but is instead being converted to the membrane lipid PC. The native canola *LPCAT* lacks the capability to handle MCFAs (Zhang *et al*., 2015), so the route to PC could be via *PDCT* or *CPT* exchange. Consequently, this inefficient utilization of laurate for TAG synthesis was also associated with a negatively correlated penalty in total oil yields (Knutzon *et al*., 1999).

Similar to the expression of MCFA in seed oil, the over‐expression of MCFA in the leaf high oil model (Vanhercke *et al*., 2013) with the co‐expression of *CnGPAT9* and *CnLPAAT* there is a metabolic bottleneck identified with the sequestering of MCFA in PC. It appeared that the *CnGPAT9* does not exhibit substrate specificities, but rather actively incorporates acyl‐CoAs based on their availability, although this requires further investigation. The low abundance of MCFA‐containing DAG species suggests that *de novo* DAG is quickly converted to PC through the activities of *PDCT* or *CPT*, due to the absence of a substrate‐specific DGAT capable of using the MCFA‐containing DAG for TAG assembly. It was hypothesized that the addition of a *DGAT1* with substrate specificity for MCFA is essential to relieve this bottleneck and hence promote synthesis of MCFA‐enriched TAG. Previously, it has been demonstrated that *PDAT* is also involved in the maintenance of membrane homoeostasis, through the removal of damaged or unusual fatty acids from the membrane lipids and sequestering them into TAG (Fan *et al*., 2013, 2014). This suggests that the expression of a substrate‐specific PDAT may have assisted with the recovery of the observed phenotype. This study demonstrated that the over‐expression of the *DGAT1* from *E. guineensis* (*EgDGAT1*) was sufficient enough in restoring membrane homoeostasis by avoiding the initial accumulation of MCFA in PC. The expression of *EgDGAT1* proved that a substrate‐specific *DGAT* is essential for the efficient assembly of TAG, through the relationship of decreased PC species and increased TAG species containing MCFA.

The addition of *EgDGAT1* plays an important role in the assembly of TAG, particularly those containing C12:0 and C14:0, associated with expression of *UcTE* and *CcTE*, respectively. The increased amounts of tri‐MCFA TAG species, compared to the samples expressing only the *CnGPAT9* and *CnLPAAT*, highlights the importance of expressing an appropriate *DGAT* for efficient TAG assembly. Collectively, these results highlight an efficient assembly pathway for the incorporation of MCFA into plant oils, while also efficiently avoiding accumulation in membrane lipids. We hypothesized that through the relief of the metabolic bottleneck of PC accumulation, the complete assembly pathway assists in promoting higher oil yields. This was supported by the observed increases in both TFA and TAG contents following *EgDGAT1* expression, and hence achieved via preventing inhibition of the FAS cycle previously observed with an incomplete pathway for the utilization of MCFA‐CoA (Knutzon *et al*., 1999; Wiberg *et al*., 1997). This is also supported by the results of previous studies that have demonstrated that the inefficient utilization of unusual fatty acids for the assembly of glycerolipids is correlated with both their accumulation in phospholipids (Knutzon *et al*., 1999) and the inhibition of fatty acid synthesis (Bates *et al*., 2014).

It is suspected that the concept of substrate‐specific DGATs could be further extended into the work of improving the accumulation of other unusual fatty acids, such as short‐chain, epoxy and hydroxyl fatty acids, in both seed and plant biomass platforms. The identification of a substrate‐specific *DGAT* followed by its over‐expression in the system of interest should assist in the incorporation of the respective fatty acid into TAG. Another alternative for improving the accumulation of unusual fatty acids could be the modification of the active site through methods such as site‐directed mutagenesis (Liu *et al*., 2010; Morand *et al*., 1998) and hence modify or improve the substrate specificity of acyltransferases. For example, the *EgDGAT1* exhibited some specificity for C14:0, but preferred C12:0 and C16:0. It may be possible to modify the active site of the *EgDGAT1* to explicitly utilize C14:0 for incorporation into TAG. Although in this study significant levels of C14:0 incorporation into TAG was achieved, with ≈40%, the addition of an engineered *DGAT* with explicit substrate specificity for C14:0 should contribute to increasing the myristate content of TAG.

It has been well established that thioesterases are an important factor in the determination of the fatty acid composition of TAG (Bates *et al*., 2013; Dehesh *et al*., 1996a; Harwood, 1996). Historically, transgenic work has been associated with the over‐expression of single thioesterases (Dehesh *et al*., 1996b; Dörmann *et al*., 2000; Eccleston *et al*., 1996; Leonard *et al*., 1998), for the modification of TAG composition. In this study, different combinations of thioesterases were studied to investigate the potential shift in the MCFA composition of TAG and also highlight the contribution that thioesterases have towards determining and modifying the oil composition of both the native species and transgenic plants, respectively. It was demonstrated that the TAG fatty acid composition can be significantly altered through the co‐expression of thioesterases with different substrate specificities, where they work together to determine the final lipid profile. This occurrence could be explained through an increased variety of acyl‐CoAs that are available for TAG assembly and hence demonstrating that thioesterases are an integral factor in the determination of the fatty acid profile of plant oils. To efficiently utilize the increased variety within the acyl‐CoA pool for TAG assembly, it may also be necessary to over‐express various *DGAT*s with suitable substrate specificity.

It has long been considered that the expression of a *DGAT1* plays a significant role in determining plant oil compositions (Bates and Browse, 2012). Importantly, the results we present indicate that the expression of a substrate‐specific *DGAT1* is essential in the avoidance of bottleneck accumulation of MCFA in PC and hence directing the flux of MCFA to TAG production. This is achieved through the efficient utilization of the MCFA‐CoAs, produced by the termination of fatty acid synthesis by thioesterase expression, for the synthesis of TAG from *de novo* DAG. This reconfigured Kennedy pathway for improving MCFA incorporation into TAG has the potential to be further explored in both seed and leaf oil contexts and may potentially be applied for improving the accumulation of other unusual fatty acids. Of particular importance, the identification of an appropriate *DGAT* for the fatty acid profile of interest (MCFA or other unusual fatty acids) is essential for achieving increased oil production with specifically tailored fatty acid compositions.

## Methods

### Gene selection

Thioesterases were identified from previous publications, with codon optimized gene coding sequences being synthesized (Geneart, Regensburg, Germany) for *Cinnamomum camphora* 14:0‐ACP thioesterase (referred to as ‘*CcTE’*, Q39473.1 (Yuan *et al*., 1995)), *Umbellularia californica* 12:0‐ACP thioesterase (*UcTE*, Q41635.1 (Voelker *et al*., 1992), *Cocos nucifera* acyl‐ACP thioesterase *FatB2* (*CnTE2*, AEM72520.1 (Jing *et al*., 2011)). The *C. nucifera LPAAT* (*CnLPAAT*, Q42670.1 (Knutzon *et al*., 1995)) was also synthesized. *WRI1* and *DGAT1* expression vectors were produced as previously described by Vanhercke *et al*. (2013).

From a recently published transcriptome of African oil palm (*Elaeis guineensis*) (Dussert *et al*., 2013), three *DGAT* candidates were chosen for testing their capability of efficiently utilizing MCFA for the assembly of leaf lipids. The *DGAT*s from oil palm were selected based on the fatty acid compositions of palm oil and palm kernel oil (Edem, 2002), being high in MCFA content.

The glycerol‐3‐phosphate acyltransferase 9 from *C. nucifera* (*CnGPAT9*) was identified from a recently constructed transcriptome, constructed from RNA isolated from developing coconut endosperm. Using the recently identified and confirmed *Arabidopsis thaliana GPAT9* (*AtGPAT9*) (Shockey *et al*., 2016) as the BLAST query, a good candidate for *GPAT9* from coconut was identified. The *CnGPAT9* (NCBI accession number: KX235871) was identified based on sequence homology with *AtGPAT9*. Using the synthesized coconut cDNA as template, Phusion high fidelity (New England Biolabs, Hitchin, England) PCR was performed (according to manufacturer's instructions) to attempt the amplification of the full length *CnGPAT9* sequence.

### Construct assembly

Each gene was cloned into the *Eco*RI site of a binary vector, pJP3343, which already contained a 35S promoter with duplicated enhancer region (Vanhercke *et al*., 2013). *Agrobacterium tumefaciens* strain AGL1 was transformed with each of the constructs.

### *Nicotiana benthamiana* transient assay

Transient expression in *N. benthamiana* leaves was performed as previously described (Wood *et al*., 2009), with some minor modifications. *A. tumefaciens* cultures containing the gene coding for the p19 viral suppressor protein and the chimeric gene(s) of interest were mixed such that the final OD_600_ of each culture was equal to 0.1 prior to infiltrations. Each complete experiment was repeated five times to confirm reproducibility, although the data were presented for only a single experiment. For fatty acid profile analyses, a total of 16 leaves from six plants were infiltrated with the different gene combinations. The samples being compared were randomly located, with a p19 control infiltrated for each plant. After infiltrations, the *N. benthamiana* plants were grown for a further 5 days before leaf discs were harvested, freeze‐dried, weighed and stored at −80 °C.

### Total lipid extraction and fatty acid profile analysis

Total lipids were extracted from freeze‐dried *N. benthamiana* leaves. During the extraction of total lipids, TAG 51:0 (tri‐C17:0) was added as the internal standard for the quantification of both the TAG and total fatty acid (TFA) contents. Freeze‐dried leaf tissue was ground to powder in a microcentrifuge tube containing a metallic ball using Reicht tissue lyser (Qiagen) for 3 min. at 20 frequency/s. Chloroform:methanol (2:1, v/v) was added and mixed for a further 3 min. on the tissue lyser before the addition of 1:3 (v/v) of 0.1 m KCl. The sample was then mixed for a further 3 min. before centrifugation (5 min. at 14 000 ***g***), after which the lower lipid phase was collected. The remaining phase was washed once with chloroform, and the lower phase extracted and pooled with the earlier extract. Lipid phase solvent was then evaporated completely using N_2_ gas flow and the lipids resuspended in 5 μL chloroform per mg of original dry leaf weight.

Fatty acid methyl esters (FAMEs) of total lipids (equivalent to 10 mg dry weight) were produced by incubating extracted lipid in 1 N methanolic HCl (Supelco, Bellefonte, PA) at 80 °C for 3 h. FAMEs were analysed by an Agilent 7890A gas chromatograph coupled with flame ionization detector (GC‐FID, Agilent Technologies, Palo Alto, CA), on a BPX70 column (30 m, 0.25 mm inner diameter, 0.25 μm film thickness, SGE) essentially as described previously (Zhou *et al*., 2011), except the column temperature programme. The column temperature was programmed as an initial temperature at 100 °C holding for 3 min, ramping to 240 °C at a rate of 7 °C/min and holding for 1 min. NuChek GLC‐426 was used as the external reference standard. Peaks were integrated with Agilent Technologies ChemStation software (Rev B.04.03 (16)).

### TLC analysis

From the total lipid extracts (equivalent to 10 mg dry weight), TAG and polar lipids were fractionated by TLC (Silica gel 60, MERCK) in hexane:diethylether:acetic acid (70:30:1 v/v/v) and visualized by spraying Primuline (Sigma, 5 mg/100 mL acetone:water (80:20 v/v)) and exposing plate under UV. TLC analysis was primarily used for the identification of fatty acid composition of TAG and phospholipids from lipid extraction samples. This also enabled the determination of the total TAG content for each sample. The TAG and phospholipid fractions were scraped from the TLC plates and methylated according to the FAME preparation protocol described previously.

### LC‐MS analysis

Lipids extracted from 1 mg dry leaf weight were resuspended and diluted to 1 mg/mL in mL butanol:methanol (1:1, v/v) and analysed by liquid chromatography–mass spectrometry (LC‐MS), based on previously described methods (Petrie *et al*., 2012). Briefly, lipids were chromatographically separated using a Waters BEH C8 (100 × 2.1 mm, 2.7 μm) fitted to an Agilent 1290 series LC and 6490 triple quadrupole LC‐MS with Jet Stream ionization with a binary gradient flow rate of 0.2 mL/min. The mobile phases were as follows: A. H_2_O:acetonitrile (10:90, v/v) with 10 mm ammonium formate and 0.2% acetic acid; B. H_2_O:acetonitrile:isopropanol (5:15:80, v/v) with 10 mm ammonium formate and 0.2% acetic acid. For the phosphatidylcholine (PC) and lysophosphatidylcholine (LPC), species hydrogen adducts were quantified by the characteristic 184 *m/z* phosphatidyl head group ion under positive ionization mode. The ammonium adducts of monogalactosyl diacylglycerol (MGDG), digalactosyl diacylglycerol (DGDG), diacylglycerol (DAG) and TAG lipid species were analysed by the neutral loss of singular fatty acids C_12_ to C_18_. Multiple reaction monitoring (MRM) lists were based on the following major fatty acids: 12:0, 14:0, 16:0, 16:3, 18:0, 18:1, 18:2, 18:3, using a collision energy of 28 V for all lipid classes except for DAG where a collision energy of 14 V was used. Individual MRM TAG was identified based on ammoniated precursor ion and product ion from neutral loss.

### Statistical analysis

To determine whether there was any significant differences between samples, statistical analysis was performed using either the data analysis package for Excel 2013 or the dplyr, ggplot2 packages from R using RStudio (Horton and Kleinman, 2015). Statistical analyses were performed using students *T*‐test, for two samples assuming unequal variances. The experiments were designed with either three or four replicates, to enable accurate analysis of any significant differences.

## Conflict of interest

We confirm that this work is original and has not been published elsewhere nor is there any associated conflict of interest.

## Supporting information


**Figure S1.** Complete protein sequence alignment of GPAT9 from *Arabidopsis thaliana* (*At*GPAT9) (Shockey *et al*., 2016) and *Cocos nucifera* (*Cn*GPAT9) identified from transcriptome.
**Figure S2.** Phylogenetic relationship of glycerol‐3‐phosphate acyltransferase (*GPAT*) genes from various species.
**Figure S3.** Investigating the accumulation and assembly of MCFA in plant leaf lipids, testing different *DGAT1* candidates, with representative images shown for each treatment (*n* = 3).
**Figure S4.** Analysis of *diacylglycerol acyltransferase 1* (*DGAT1*) protein sequences by multiple sequence alignment, investigating differences in *Arabidopsis thaliana* (*AtDGAT1*; NP_179535) and predicted *DGAT1* isoforms from *Elaeis guineensis* (Dussert *et al*., 2013).
**Figure S5.** Photos of *Nicotiana benthamiana* transient study, investigating the reconstruction of the Kennedy pathway tailored for improving the accumulation of MCFA, with representative images shown for each treatment (*n* = 3).
**Figure S6.** Analysis of diacylglycerol (DAG) lipid species, associated with the expression of thioesterase from *Umbellularia californica* (UcTE), *Cinnamomum camphora* (CcTE) or *Cocos nucifera* (CnTE2) (*n* = 3). Control refers to samples that were p19 only infiltrations. CnGPAT9 = *C. nucifera* glycerol‐3‐phosphate acyltransferase 9; CnLPAAT = *C. nucifera* lysophosphatidic acid acyltransferase; EgDGAT1 = *Elaeis guineensis* diacylglycerol acyltransferase.
**Figure S7.** Following *Nicotiana benthamiana* infiltrations testing different combinations of thioesterases, the triacylglycerol (TAG) species profile was investigated via LC‐MS.
