# Supplementary material for: A reconfigured Kennedy pathway which promotes efficient accumulation of medium‐chain fatty acids in leaf oils
Source: Plant Biotechnol J. 2017 May 3;15(11):1397–408. doi: 10.1111/pbi.12724 (PMC5633779; doi:10.1111/pbi.12724)
Supplement: Supplementary file 1 — Figure S1. Complete protein sequence alignment of GPAT9 from Arabidopsis thaliana (AtGPAT9) (Shockey et al., 2016) and Cocos nucifera (CnGPAT9) identified from transcriptome. Figure S2. Phylogenetic relationship of glycerol‐3‐phosphate acyltransferase (GPAT) genes from various species. Figure S3. Investigating the accumulation and assembly of MCFA in plant leaf lipids, testing different DGAT1 candidates, with representative images shown for each treatment (n = 3). Figure S4. Analysis of diacylglycerol acyltransferase 1 (DGAT1) protein sequences by multiple sequence alignment, investigating differences in Arabidopsis thaliana (AtDGAT1; NP_179535) and predicted DGAT1 isoforms from Elaeis guineensis (Dussert et al., 2013). Figure S5. Photos of Nicotiana benthamiana transient study, investigating the reconstruction of the Kennedy pathway tailored for improving the accumulation of MCFA, with representative images shown for each treatment (n = 3). Figure S6. Analysis of diacylglycerol (DAG) lipid species, associated with the expression of thioesterase from Umbellularia californica (UcTE), Cinnamomum camphora (CcTE) or Cocos nucifera (CnTE2) (n = 3). Control refers to samples that were p19 only infiltrations. CnGPAT9 = C. nucifera glycerol‐3‐phosphate acyltransferase 9; CnLPAAT = C. nucifera lysophosphatidic acid acyltransferase; EgDGAT1 = Elaeis guineensis diacylglycerol acyltransferase. Figure S7. Following Nicotiana benthamiana infiltrations testing different combinations of thioesterases, the triacylglycerol (TAG) species profile was investigated via LC‐MS. [file PBI-15-1397-s001.docx]

**Supplementary Figures**

[**Supplementary Figure 1.** Complete protein sequence alignment of GPAT9 from *Arabidopsis thaliana* (*At*GPAT9) [1] and *Cocos nucifera* (*Cn*GPAT9) identified from transcriptome.](#_Toc474515845)

[**Supplementary Figure 2.** Phylogenetic relationship of glycerol-3-phosphate acyltransferase (*GPAT*) genes from various species. The *GPAT9* genes, containing the *Arabidopsis thaliana* (*AtGPAT9*) and *Cocos nucifera* (*CnGPAT9*) genes used in this study, highlighted in green. The plant GPAT9 cluster is shaded in grey, which demonstrated a greater degree of similarity to the *Mus musculus* (mouse) homologues (shaded in green). BrGPAT3 = *Brassica rapa* glycerol-3-phosphate acyltransferase 3-like (Accession: XM_009105753); BnGPAT3 = *Brassica napus* glycerol-3-phosphate acyltransferase 3-like (Accession: XM_013896062); CsGPAT3 = *Camelina sativa* glycerol-3-phosphate acyltransferase 3 (Accession: XM_010458322); AtGPAT9 = *A. thaliana* glycerol-3-phosphate acyltransferase 9 (Accession: NM_125455); ThGPAT3 = *Tarenaya hassleriana* glycerol-3-phosphate acyltransferase 3-like (Accession: XM_010549847); RcGPAT3 = *Ricinus communis* glycerol-3-phosphate acyltransferase 3 (Accession: NM_001323761); JcGPAT3 = *Jatropha curcas* glycerol-3-phosphate acyltransferase 3 (Accession: NM_001308751); EgGPAT3 = *Elaeis guineensis* glycerol-3-phosphate acyltransferase 3-like (Accession: XM_010913693); CnGPAT9 = *C. nucifera* GPAT9 (Accession: KX235871); Mouse GPAT = Mus musculus 1-acylglycerol-3-phosphate O-acyltransferase 9 (Accession: NM_172715); LrGPAT = *Lilium regale* GPAT (Accession: JX524740); LpGPAT = *Lilium pensylvanicum* GPAT (Accession: JX524741); LlGPAT = *Lilium longiflorum* GPAT (Accession: JX524738); EgGPAT mRMA = *E. guineensis* mRNA for acylation enzyme (Accession: AJ272082); ChGPAT = *Corylus heterophylla* GPAT (Accession: JF428134); JcGPAT = *J. curcas* glycerol-3-phosphate acyltransferase, chloroplastic (Accession: NM_001305998); BnGPAT = *B. napus* glycerol-3-phosphate acyltransferase gene (Accession: KM243174); PsGPAT = *Pisum sativum* chloroplast mRNA for acyl-ACP:sn-glycerol-3-phosphate-acyltransferase (Accession: X59041); SlGPAT = *Solanum lycopersicum* glycerol-3-phosphate acyltransferase (Accession: NM_001306067); AtGPAT3 = *A. thaliana* putative sn-glycerol-3-phosphate 2-O-acyltransferase (Accession: NM_116426); AtGPAT2 = *A. thaliana* glycerol-3-phosphate sn-2-acyltransferase 2 (Accession: NM_100120); AtGPAT1 = *A. thaliana* sn-glycerol-3-phosphate 2-O-acyltransferase (Accession: NM_100531); AtGPAT7 = *A. thaliana* glycerol-3-phosphate acyltransferase 7 (Accession: NM_120691); AtGPAT5 = *A. thaliana* glycerol-3-phosphate acyltransferase 5 (Accession: NM_111976); QsGPAT = *Quercus suber* glycerol-3-phosphate acyltransferase (Accession: JN819185); EgGPAT5 = *E. guineensis* glycerol-3-phosphate acyltransferase 5 (Accession: XM_010923983); EgGPAT6 = *E. guineensis* glycerol-3-phosphate 2-O-acyltransferase 6 (Accession: XM_010924793); AtGPAT6 = *A. thaliana* bifunctional sn-glycerol-3-phosphate 2-O-acyltransferase/phosphatase (Accession: NM_129367); AtGPAT8 = *A. thaliana* bifunctional sn-glycerol-3-phosphate 2-O-acyltransferase/phosphatase (Accession: NM_116264); AtGPAT4 = *A. thaliana* glycerol-3-phosphate sn-2-acyltransferase (Accession: NM_100043); GhGPAT = *Gossypium hirsutum* probable glycerol-3-phosphate acyltransferase 3 (Accession: XM_016838669); EgGPAT4 = *E. guineensis* glycerol-3-phosphate 2-O-acyltransferase 4-like (Accession: XM_010942191).](#_Toc474515846)

[**Supplementary Figure 3.** Investigating the accumulation and assembly of MCFA in plant leaf lipids, testing different *DGAT1* candidates, with representative images shown for each treatment (n=3). Treatment combinations and corresponding photos of *Nicotiana benthamiana* leaves, following five days of gene expression. The label “TE” represents the expression of the thioesterase from *Cinnamomum camphora* (*CcTE*). CnLPAAT = *Cocos nucifera* lysophosphatidic acid acyltransferase; AtWRI1 = *Arabidopsis thaliana* WRINKLED1; AtDGAT1 = *A. thaliana* diacylglycerol acyltransferase 1; EgDGAT = *Elaeis guineensis* diacylglycerol acyltransferase.](#_Toc474515847)

[**Supplementary Figure 4.** Analysis of *diacylglycerol acyltransferase 1* (*DGAT1*) protein sequences by multiple sequence alignment, investigating differences in *Arabidopsis thaliana* (*AtDGAT1*; NP_179535) and predicted *DGAT1* isoforms from *Elaeis guineensis* [2]. The sequences highlighted by the red boxes indicate the highly conserved catalytic and regulatory motifs of DGAT1 proteins [3].](#_Toc474515848)

[**Supplementary Figure 5.** Photos of *Nicotiana benthamiana* transient study, investigating the reconstruction of the Kennedy pathway tailored for improving the accumulation of MCFA, with representative images shown for each treatment (n=3). *CcTE* = *Cinnamomum camphora* thioesterase; *ClTE* = *Cuphea lanceolata* thioesterase; *UcTE* = *Umbellularia californica* thioesterase; *CnTE2* = *Cocos nucifera* thioesterase; CnGPAT9 = *C. nucifera* glycerol-3-phosphate acyltransferase 9; CnLPAAT = *C. nucifera* lysophosphatidic acid acyltransferase; *At*WRI = *Arabidopsis thaliana WRINKLED1*; *Eg*DGAT1 = *Elaeis guineensis* diacylglycerol acyltransferase 1.](#_Toc474515849)

[**Supplementary Figure 6.** Analysis of diacylglycerol (DAG) lipid species, associated with the expression of thioesterase from *Umbellularia californica* (*UcTE*), *Cinnamomum camphora* (*CcTE*) or *Cocos nucifera* (*CnTE2*) (n=3). Control refers to samples that were p19 only infiltrations. *Cn*GPAT9 = *C. nucifera* glycerol-3-phosphate acyltransferase 9; *Cn*LPAAT = *C. nucifera* lysophosphatidic acid acyltransferase; *Eg*DGAT1 = *Elaeis guineensis* diacylglycerol acyltransferase.](#_Toc474515850)

[**Supplementary Figure 7.** Following *Nicotiana benthamiana* infiltrations testing different combinations of thioesterases, the triacylglycerol (TAG) species profile was investigated via LC-MS. Panels are grouped for each thioesterase combination (n=4). The “Control” refers to the infiltration of p19 alone. The label “Thioesterase Only” refers to the expression of p19 with the respective thioesterase combination. The label “*At*WRI1+*Cn*LPAAT+*Eg*DGAT1” refers to the expression of *Arabidopsis thaliana WRINKLED1* (*At*WRI1), *Cocos nucifera* *lysophosphatidic acid acyltransferase* (*Cn*LPAAT) and *Elaeis guineensis* *diacylglycerol acyltransferase* (*Eg*DGAT1), in combination with the respective thioesterase combinations. *Cc*TE = *Cinnamomum camphora* thioesterase; *Cn*TE = *C. nucifera* thioesterase; *Uc*TE = *Umbellularia californica* thioesterase.](#_Toc474515851)

# **Supplementary Data**

## Supplementary Data

*At*GPAT9 (1) MSSTAGRLVTSKSELDLDHPNIEDYLPSGSSINEPRGKLSLRDLLDISPT

*Cn*GPAT9 (1) ----MVGLRSSSSEMDLDRPNIEEYLTTDSIQESPK-KLHLRDLLDISPT

*At*GPAT9 (51) LTEAAGAIVDDSFTRCFKSNPPEPWNWNIYLFPLYCFGVVVRYCILFPLR

*Cn*GPAT9 (46) LTEATGAIVDDSFTRCFKSNPPEPWNWNVYLFPLWCLGVIIRYGILFPLR

*At*GPAT9 (101) CFTLAFGWIIFLSLFIPVNALLKGQDRLRKKIERVLVEMICSFFVASWTG

*Cn*GPAT9 (96) VAILTAGWLVFFAAFIPVHFLLTAHNKWRRKIERKLVEMICSVFVASWTG

*At*GPAT9 (151) VVKYHGPRPSIRPKQVYVANHTSMIDFIVLEQMTAFAVIMQKHPGWVGLL

*Cn*GPAT9 (146) VVKYHGPRPSMRPQQVFVANHTSMIDFIILEQMTAFAVIMQKHPGWVGFI

*At*GPAT9 (201) QSTILESVGCIWFNRSEAKDREIVAKKLRDHVQGADSNPLLIFPEGTCVN

*Cn*GPAT9 (196) QKTILEGVGCIWFNRTESKDREVVARKLREHIHGADNNPLLIFPEGTCVN

*At*GPAT9 (251) NNYTVMFKKGAFELDCTVCPIAIKYNKIFVDAFWNSRKQSFTMHLLQLMT

*Cn*GPAT9 (246) NHYTVMFKKGAFELGCAVCPVAIKYNKIFVDAFWNSKKQSFTMHLFHLMT

*At*GPAT9 (301) SWAVVCEVWYLEPQTIRPGETGIEFAERVRDMISLRAGLKKVPWDGYLKY

*Cn*GPAT9 (296) SWAVVCDVWYLEPQYIRPGETPIEFAERVRDMISVRAGLKKVPWDGYLKY

*At*GPAT9 (351) SRPSPKHSERKQQSFAESILARLEEK

*Cn*GPAT9 (315) FRPSPKLTERKQQIFAESVLQRLEEK

Supplementary Figure 1. Complete protein sequence alignment of GPAT9 from *Arabidopsis thaliana* (*At*GPAT9) [1] and *Cocos nucifera* (*Cn*GPAT9) identified from transcriptome.

**
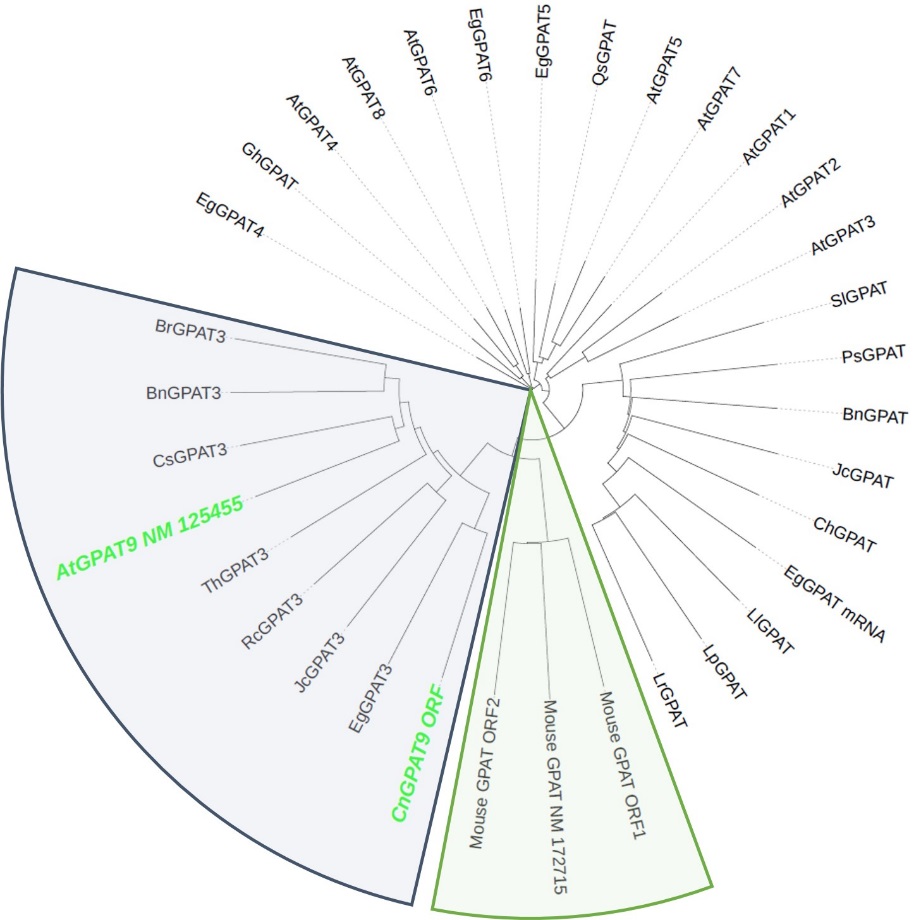
**

Supplementary Figure 2. Phylogenetic relationship of glycerol-3-phosphate acyltransferase (*GPAT*) genes from various species. The *GPAT9* genes, containing the *Arabidopsis thaliana* (*AtGPAT9*) and *Cocos nucifera* (*CnGPAT9*) genes used in this study, highlighted in green. The plant GPAT9 cluster is shaded in grey, which demonstrated a greater degree of similarity to the *Mus musculus* (mouse) homologues (shaded in green).
BrGPAT3 = *Brassica rapa* glycerol-3-phosphate acyltransferase 3-like (Accession: XM_009105753); BnGPAT3 = *Brassica napus* glycerol-3-phosphate acyltransferase 3-like (Accession: XM_013896062); CsGPAT3 = *Camelina sativa* glycerol-3-phosphate acyltransferase 3 (Accession: XM_010458322); AtGPAT9 = *A. thaliana* glycerol-3-phosphate acyltransferase 9 (Accession: NM_125455); ThGPAT3 = *Tarenaya hassleriana* glycerol-3-phosphate acyltransferase 3-like (Accession: XM_010549847); RcGPAT3 = *Ricinus communis* glycerol-3-phosphate acyltransferase 3 (Accession: NM_001323761); JcGPAT3 = *Jatropha curcas* glycerol-3-phosphate acyltransferase 3 (Accession: NM_001308751); EgGPAT3 = *Elaeis guineensis* glycerol-3-phosphate acyltransferase 3-like (Accession: XM_010913693); CnGPAT9 = *C. nucifera* GPAT9 (Accession: KX235871); Mouse GPAT = Mus musculus 1-acylglycerol-3-phosphate O-acyltransferase 9 (Accession: NM_172715); LrGPAT = *Lilium regale* GPAT (Accession: JX524740); LpGPAT = *Lilium pensylvanicum* GPAT (Accession: JX524741); LlGPAT = *Lilium longiflorum* GPAT (Accession: JX524738); EgGPAT mRMA = *E. guineensis* mRNA for acylation enzyme (Accession: AJ272082); ChGPAT = *Corylus heterophylla* GPAT (Accession: JF428134); JcGPAT = *J. curcas* glycerol-3-phosphate acyltransferase, chloroplastic (Accession: NM_001305998); BnGPAT = *B. napus* glycerol-3-phosphate acyltransferase gene (Accession: KM243174); PsGPAT = *Pisum sativum* chloroplast mRNA for acyl-ACP:sn-glycerol-3-phosphate-acyltransferase (Accession: X59041); SlGPAT = *Solanum lycopersicum* glycerol-3-phosphate acyltransferase (Accession: NM_001306067); AtGPAT3 = *A. thaliana* putative sn-glycerol-3-phosphate 2-O-acyltransferase (Accession: NM_116426); AtGPAT2 = *A. thaliana* glycerol-3-phosphate sn-2-acyltransferase 2 (Accession: NM_100120); AtGPAT1 = *A. thaliana* sn-glycerol-3-phosphate 2-O-acyltransferase (Accession: NM_100531); AtGPAT7 = *A. thaliana* glycerol-3-phosphate acyltransferase 7 (Accession: NM_120691); AtGPAT5 = *A. thaliana* glycerol-3-phosphate acyltransferase 5 (Accession: NM_111976); QsGPAT = *Quercus suber* glycerol-3-phosphate acyltransferase (Accession: JN819185); EgGPAT5 = *E. guineensis* glycerol-3-phosphate acyltransferase 5 (Accession: XM_010923983); EgGPAT6 = *E. guineensis* glycerol-3-phosphate 2-O-acyltransferase 6 (Accession: XM_010924793); AtGPAT6 = *A. thaliana* bifunctional sn-glycerol-3-phosphate 2-O-acyltransferase/phosphatase (Accession: NM_129367); AtGPAT8 = *A. thaliana* bifunctional sn-glycerol-3-phosphate 2-O-acyltransferase/phosphatase (Accession: NM_116264); AtGPAT4 = *A. thaliana* glycerol-3-phosphate sn-2-acyltransferase (Accession: NM_100043); GhGPAT = *Gossypium hirsutum* probable glycerol-3-phosphate acyltransferase 3 (Accession: XM_016838669); EgGPAT4 = *E. guineensis* glycerol-3-phosphate 2-O-acyltransferase 4-like (Accession: XM_010942191).


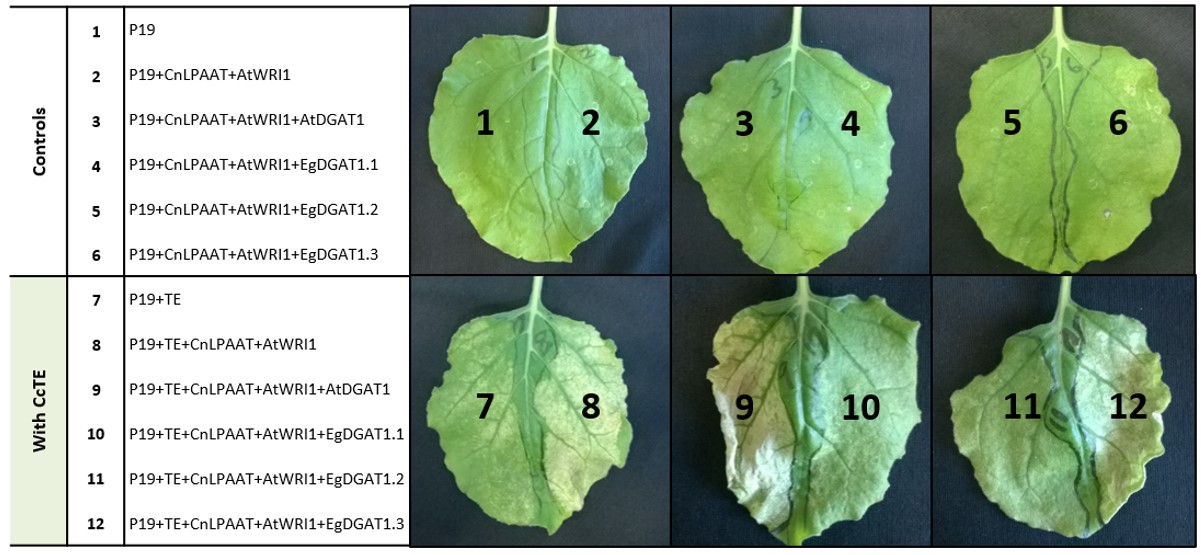


Supplementary Figure 3. Investigating the accumulation and assembly of MCFA in plant leaf lipids, testing different *DGAT1* candidates, with representative images shown for each treatment (n=3). Treatment combinations and corresponding photos of *Nicotiana benthamiana* leaves, following five days of gene expression. The label “TE” represents the expression of the thioesterase from *Cinnamomum camphora* (*CcTE*). CnLPAAT = *Cocos nucifera* lysophosphatidic acid acyltransferase; AtWRI1 = *Arabidopsis thaliana* WRINKLED1; AtDGAT1 = *A. thaliana* diacylglycerol acyltransferase 1; EgDGAT = *Elaeis guineensis* diacylglycerol acyltransferase.


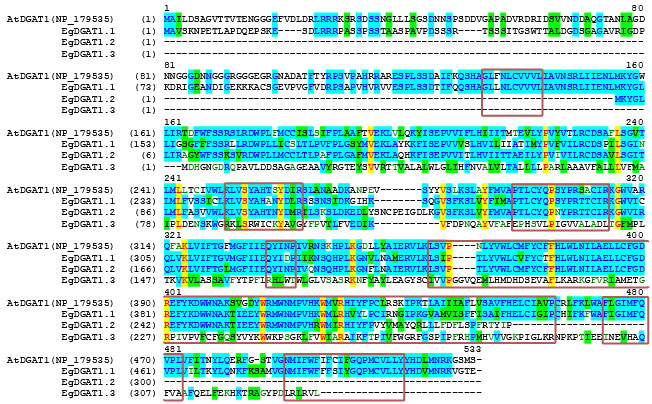


Supplementary Figure 4. Analysis of *diacylglycerol acyltransferase 1* (*DGAT1*) protein sequences by multiple sequence alignment, investigating differences in *Arabidopsis thaliana* (*AtDGAT1*; NP_179535) and predicted *DGAT1* isoforms from *Elaeis guineensis* [2]. The sequences highlighted by the red boxes indicate the highly conserved catalytic and regulatory motifs of DGAT1 proteins [3].


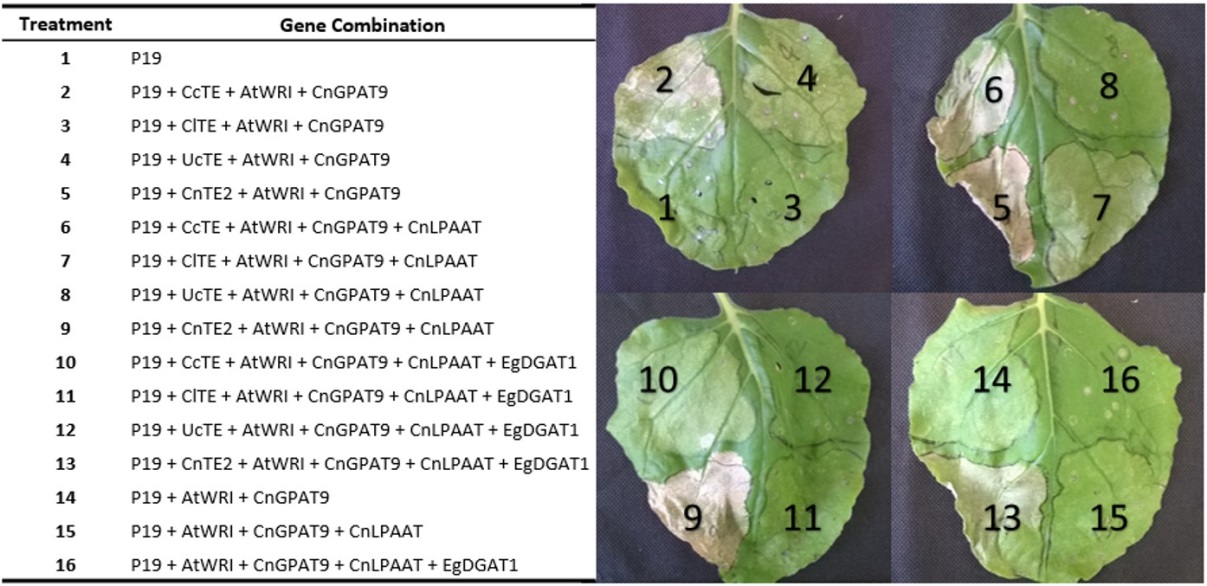


Supplementary Figure 5. Photos of *Nicotiana benthamiana* transient study, investigating the reconstruction of the Kennedy pathway tailored for improving the accumulation of MCFA, with representative images shown for each treatment (n=3). *CcTE* = *Cinnamomum camphora* thioesterase; *ClTE* = *Cuphea lanceolata* thioesterase; *UcTE* = *Umbellularia californica* thioesterase; *CnTE2* = *Cocos nucifera* thioesterase; CnGPAT9 = *C. nucifera* glycerol-3-phosphate acyltransferase 9; CnLPAAT = *C. nucifera* lysophosphatidic acid acyltransferase; *At*WRI = *Arabidopsis thaliana WRINKLED1*; *Eg*DGAT1 = *Elaeis guineensis* diacylglycerol acyltransferase 1.


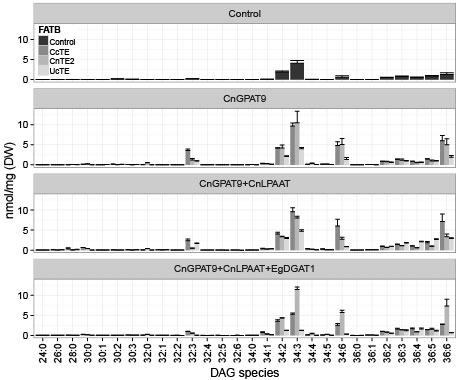


Supplementary Figure 6. Analysis of diacylglycerol (DAG) lipid species, associated with the expression of thioesterase from *Umbellularia californica* (*UcTE*), *Cinnamomum camphora* (*CcTE*) or *Cocos nucifera* (*CnTE2*) (n=3). Control refers to samples that were p19 only infiltrations. *Cn*GPAT9 = *C. nucifera* glycerol-3-phosphate acyltransferase 9; *Cn*LPAAT = *C. nucifera* lysophosphatidic acid acyltransferase; *Eg*DGAT1 = *Elaeis guineensis* diacylglycerol acyltransferase.


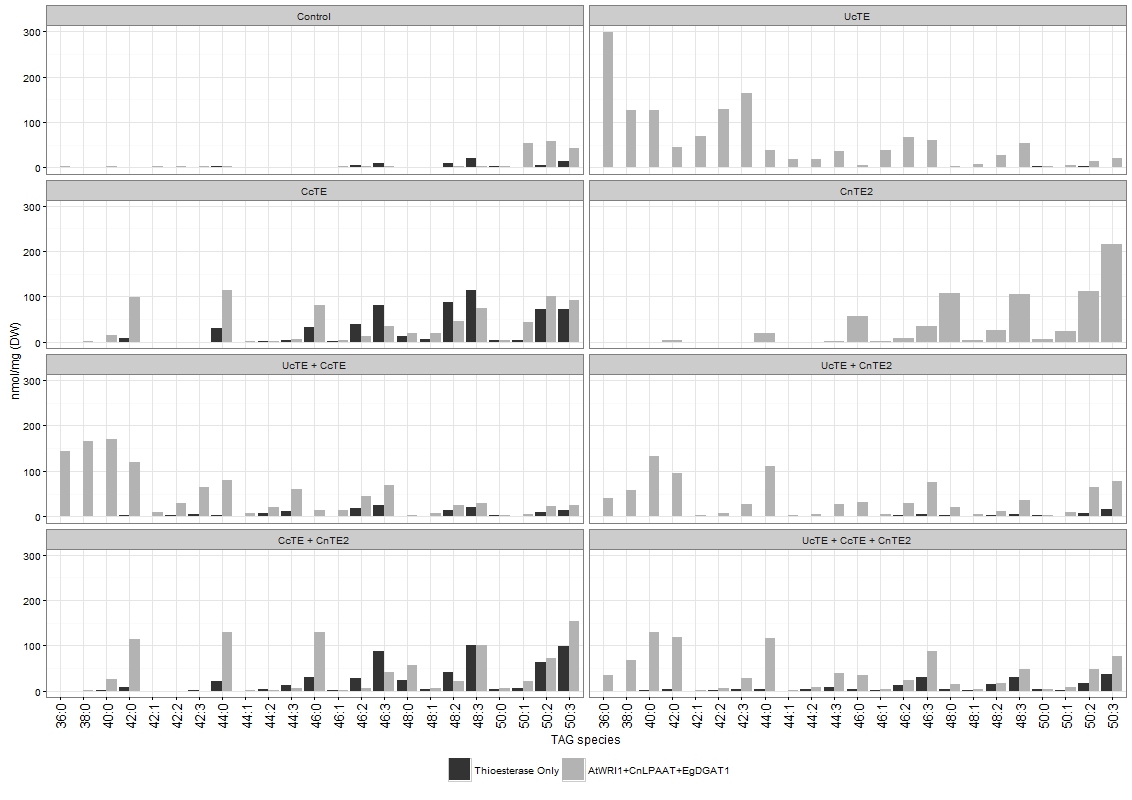


Supplementary Figure 7. Following *Nicotiana benthamiana* infiltrations testing different combinations of thioesterases, the triacylglycerol (TAG) species profile was investigated via LC-MS. Panels are grouped for each thioesterase combination (n=4). The “Control” refers to the infiltration of p19 alone. The label “Thioesterase Only” refers to the expression of p19 with the respective thioesterase combination. The label “*At*WRI1+*Cn*LPAAT+*Eg*DGAT1” refers to the expression of *Arabidopsis thaliana WRINKLED1* (*At*WRI1), *Cocos nucifera* *lysophosphatidic acid acyltransferase* (*Cn*LPAAT) and *Elaeis guineensis* *diacylglycerol acyltransferase* (*Eg*DGAT1), in combination with the respective thioesterase combinations. *Cc*TE = *Cinnamomum camphora* thioesterase; *Cn*TE = *C. nucifera* thioesterase; *Uc*TE = *Umbellularia californica* thioesterase.

1. Shockey, J., Regmi, A., Cotton, K., Adhikari, N., Browse, J. & Bates, P. D. (2016) Identification of Arabidopsis GPAT9 (At5g60620) as an Essential Gene Involved in Triacylglycerol Biosynthesis, *Plant Physiol.* **170**, 163-79.

2. Dussert, S., Guerin, C., Andersson, M., Joet, T., Tranbarger, T. J., Pizot, M., Sarah, G., Omore, A., Durand-Gasselin, T. & Morcillo, F. (2013) Comparative transcriptome analysis of three oil palm fruit and seed tissues that differ in oil content and fatty acid composition, *Plant Physiol.* **162**, 1337-58.

3. Cao, H. (2011) Structure-function analysis of diacylglycerol acyltransferase sequences from 70 organisms, *BMC research notes.* **4**, 249.
